# Sodium danshensu attenuates cerebral ischemia–reperfusion injury by targeting AKT1

**DOI:** 10.3389/fphar.2022.946668

**Published:** 2022-09-15

**Authors:** Qing Gao, Hao Deng, Zhengfei Yang, Qiuyue Yang, Yilin Zhang, Xiaopeng Yuan, Miao Zeng, Maojuan Guo, Wenyun Zeng, Xijuan Jiang, Bin Yu

**Affiliations:** ^1^ School of Integrative Medicine, Tianjin University of Traditional Chinese Medicine, Tianjin, China; ^2^ Tianjin Key Laboratory of Translational Research of TCM Prescription and Syndrome, First Teaching Hospital of Tianjin University of Traditional Chinese Medicine, Tianjin, China; ^3^ College of Traditional Chinese Medicine, Ningxia Medical University, Yinchuan, China; ^4^ Shenzhen Traditional Chinese Medicine Hospital, Shenzhen, China; ^5^ International Exchanges Department and International Education College, Tianjin University of Traditional Chinese Medicine, Tianjin, China

**Keywords:** sodium danshensu, cerebral ischemic reperfusion injury, AKT1, drug target, protein array analysis

## Abstract

The beneficial properties of Sodium Danshensu (SDSS) for controlling cerebral ischemia and reperfusion injury (CIRI) are elucidated here both *in vivo* and *in vitro*. SDSS administration significantly improved the viability of P12 cells, reduced lactate dehydrogenase (LDH) leakage, and decreased the apoptosis rate following exposure to an oxygen-glucose deprivation/reoxygenation (OGD) environment. In addition, the results of a Huprot^TM^ human protein microarray and network pharmacology indicated that AKT1 is one of the main targets of SDSS. Moreover, functional experiments showed that SDSS intervention markedly increased the phosphorylation level of AKT1 and its downstream regulator, mTOR. The binding sites of SDSS to AKT1 protein were confirmed by Autodock software and a surface plasmon resonance experiment, the result of which imply that SDSS targets to the PH domain of AKT1 at ASN-53, ARG-86, and LYS-14 residues. Furthermore, knockdown of AKT1 significantly abolished the role of SDSS in protecting cells from apoptosis and necrosis. Finally, we investigated the curative effect of SDSS in a rat model of CIRI. The results suggest that administration of SDSS significantly reduces CIRI-induced necrosis and apoptosis in brain samples by activating AKT1 protein. In conclusion, SDSS exerts its positive role in alleviating CIRI by binding to the PH domain of AKT1 protein, further resulting in AKT1 activation.

## 1 Introduction

The incidence of ischemic stroke has been increasing for the past several decades. It has become the third-highest cause of death after cancer and cardiovascular diseases, and it leads to a high rate of disability (Meschia and Brott, 2018). Currently, thrombolysis is the first-line treatment for ischemic stroke, whilst antiplatelet, anticoagulation, and neuroprotection therapies are also applied (Liu et al., 2020). However, the reperfusion that follows these treatments leads to a series of pathological insults, including lipid peroxidation, inflammation, and NO release, and ultimately cerebral ischemia and reperfusion injury (CIRI) ensues (Wu et al., 2018). At present, free radical scavengers and calcium channel blockers are not effective in clinical practice (Pan et al., 2007; Bhaskar et al., 2018). One study pointed out that the antioxidants will be dwarfed by the body’s free radical defence system (Davies and Holt, 2018). Another study reported that exposure to calcium channel blockers can lead to life-threatening side effects on blood pressure, cardiac conduction, and the digestive tract and nervous system (Hayes, 2018). Therefore, it is urgent to seek and develop other effective treatments for CIRI.

The reperfusion injury salvage kinase (RISK) pathway plays a significant role in CIRI (Rossello and Yellon, 2017). It contains two cascades, phosphoinositide 3-kinase/AKT (PI3K/AKT) and mitogen-activated protein kinase/extracellular signal-regulated kinase (MEK/Erk). PI3K and MEK are initiators of the protective response following ischemic/reperfusion injury (Manning and Toker, 2017). Activation of either PI3K/AKT or MEK/Erk could prevent apoptosis by inhibiting apoptotic proteins, such as BCL2-associated agonist of cell death protein (BAD) and caspase cascades. In particular, AKT could activate the inflammation response by activating the NFκB pathway (Rai et al., 2019), which initiates a variety of survival pathways.

Sodium Danshensu (SDSS), also known as sodium 3-(3,4-dihydroxyphenyl)-2-hydroxypropanoate, has been widely used in the clinic to treat cardiovascular diseases such as myocardial infarction, atherosclerosis, and angina pectoris (Li et al., 2018; Zhang et al., 2019). SDSS shows great protective effects on myocardial ischemia and reperfusion injuries, significantly reducing the myocardium infarct sizes by 25%–50% (Yin et al., 2013). It also improves the survival of neonatal rat cardiomyocytes that have received I/R stress. A recent study reported that SDSS exerts a neuroprotective effect against CIRI via inhibition of apoptosis by activating the PI3K/AKT signal pathway (Tian et al., 2020). While these results are promising, the underlying mechanism is still in need of further elucidation. In this study, we have investigated the protective effect of SDSS on the CIRI model utilizing rats and rat-derived highly aggressive proliferating immortalized (HAPI) cells. Our results show that SDSS prevents apoptosis and enhances cell viability. We have also discovered that SDSS provides these neuroprotective effects by binding to the AKT1 protein.

## 2 Materials and methods

### 2.1 Materials and regents

SDSS was purchased from Selleck Co., Ltd. (Shanghai, China). Triphenyl tetrazolium chloride (TTC) reagents were provided by Solarbio Science & Technology Co., Ltd. (Beijing, China). An *In Situ* Cell Death Detection Kit was purchased from Roche (Shanghai, China). PC 12 and HAPI cell lines were purchased from ATCC (Beijing, China). Biotin-labeled SDSS (Bio-SDSS) and HuProt™ human protein microarray were provided by Wayen Biotechnologies, Inc. (Shanghai, China). CCK8 kits were purchased from DOJINDO (Beijing, China) and LDH ELISA detection kits were purchased from Nanjing Jiancheng Bioengineering Institute (Nanjing, China). Rabbit anti-rat antibodies, including mTOR (Lot No.2983S), phospho-mTOR (Ser 2448, Lot No.5536S), PI3K (Lot No.D55D5), AKT (Lot No. 4685S), and phospho-AKT (Ser473, Lot No. 4060S), were purchased from Cell Signaling Technology (Boston, United States of America). Rabbit anti-rat antibody GAPDH (Lot No. ab181602) was purchased from Abcam (Shanghai, China). SiRNAs were purchased from Thermo Fisher (Shanghai, China).

### 2.2 Cell culture and oxygen and glucose deprivation model

HAPI and PC12 cell lines (Passages 3–6 were used) were all cultured in DMEM-H medium (supplemented with 10% HI-FBS, 100 U/mL penicillin, and 100 μg/ml streptomycin) at 37°C in an incubator containing 5% CO_2_ and 5% O_2_, with 100% humidity. The oxygen and glucose deprivation (OGD) model was incubated in DMEM-H medium, then was replaced by HEPES medium in an incubator containing 5% CO_2_ and 1% O_2_ (with 100% humidity to achieve hypoxia) for 2 h. Reoxygenation was achieved by changing the incubation medium back to DMEM-H and incubating for 24 h and 48 h. SDSS was administered during reoxygenation, to final concentrations of either 10 μM, 20 μM, 50 μM, or 100 μM.

### 2.3 TUNEL staining, cell viability, lactate dehydrogenase leakage

TUNEL staining, a cell viability assay, and an LDH leakage assay were conducted according to the instructions of the In-situ Cell Death Detection Kit, CCK8 kit, and LDH Leakage Kit, respectively.

### 2.4 Huprot^TM^ human protein microarray and network pharmacy

The human protein microarray assay and data analysis were conducted by Wayen Biotechnologies, Inc. (Shanghai, China), according to the previously published procedure (Zhao, et al., 2019). The protein microarray chip contains 20,000 proteins. The signal-to-noise ratio (SNR) was calculated as the ratio of the foreground value to the background value. For each protein dot, an SDSS group signal vs. Bio-SDSS signal (IMean_Ratio) of ≥1.4 was identified as a positive result, i.e., SDSS could bind to this protein. Network pharmacology analysis was performed by using the following databases: PubChem Compound, Pharm Mapper, UniProt, Online Mendelian Inheritance in Man (OMIM), Gender and Development (GAD), Therapeutic Target Database (TTD), and PharmGKB.

### 2.5 Autodock

Autodock was conducted through Autodock 4.1 software. Briefly: The crystal structure of AKT protein (PDB code: 2uvm) was obtained from the Protein Data Bank. The 3D structure of SDSS was downloaded from the PubChem Compound database. The latter was initialized by adding Gasteiger charges, merging nonpolar hydrogen bonds, and setting rotatable bonds, and was then rewritten to PDBQT format. The AKT protein was added with polar hydrogen. The grid box was set to contain the entire PH domain. The docking results were visualized by using PyMOL software.

### 2.6 Synthesis of AKT1 and mutant AKT1 proteins

To construct the novel pCMV-3xflag–expressing vector, the full-length sequence of wild-type AKT1 mRNA (BC000479.2) was synthesized by a biotechnology company (Shanghai, China) and cloned into the BamHI and EcoRI sites of the pCMV-3Tag-1A vector. The mutation AKT1-K14R-R86H-N54Q was also synthesized by a biotechnology company (Shanghai, China) and cloned into the BamHI and EcoRI sites of the pCMV-3Tag-1A vector. The sequences are provided in the Supplementary Materials section. Wide-type AKT1 and mutated AKT1 protein were synthesized by the following methods, respectively: pCMV3-flag-rat with wild-type AKT1 was transfected into HEK-293 cells, while pCMV3-flag-rat with mutation AKT1-K14R-R86H-N54Q were constructed and transfected into HEK-293 cells. The protein purification procedure was conducted following a previous study (Kumar et al., 2001). Briefly, 2×10^8^ cells were harvested and lysed. The extract was loaded onto Ni-NTA superflow resin using an FPLC pump, then the AKT1 protein was purified by Agarose affinity gel, which contains anti-FLAG antibody. The purified protein was stored at 4°C and used for further study within 1 week.

### 2.7 Surface plasmon resonance analysis

The surface plasmon resonance (SPR) analysis was conducted using the GE Healthcare Biomolecule Interaction System Biacore T200. The surface of a CM-5 chip was activated by 1:1 mixture of N-hydroxysuccinimide and 1-ethyl-3-(3-dimethylamino) propyl carbodiimide, which converts carboxylates on the dextran matrix into succinamide esters. Purified AKT1 (1.0 mg/ml) was desalted and then diluted 1:20 in immobilization buffer (10 mM Na-acetate pH 4.0). AKT1 was then immobilized on the activated surface chip by amine coupling in 20 mM HEPES (pH 7.0) *via* being injected overflow for 2100 s. After AKT1 immobilization, the untreated succinamide groups were quenched by 20 mM HEPES (pH 7.0). SDSS was diluted to different concentrations before being flowed past the chips at a speed of 30 μl/min. The control chips received the same treatment, except they were treated with only immobilization buffer instead of the proteins.

### 2.8 AKT1 protein knockdown

P12 cells were seeded in a 6-well plate at a density of 2.5×10^5^ cells per well. SiRNA plasmid or negative control siRNA was transfected into the cells by Lipofectamine 2000 (2 ug/ml) agent. After incubation for 24 h, the cells were used for further study.

### 2.9 Animals

Healthy Sprague-Dawley (SD) rats aged 6–8 weeks (SPF grade, weighing 260 g–280 g) were provided by Beijing Vital River Laboratory Animal Technology Co., Ltd. (License No. SCXK 2016-0006). The experiment was carried out after 1 week of adaptive feeding under conventional experimental conditions. The raising and use of animals are in accordance with the requirements of the Animal Ethics Act of Tianjin University of Traditional Chinese Medicine. The ambient temperature was set at 24 ± 1°C, the relative humidity was set at 55 ± 5%, the light/darkness cycle was maintained on 12-h intervals, and the animals were allowed to access food and drink *ad libitum*.

### 2.10 *In vivo* cerebral ischemia and reperfusion injury model

The rat CIRI model was established according to Zea Longa’s method (Longa et al., 1989). In short: A threaded bolt with a total length of 18–22 mm was inserted into the internal carotid artery to induce cerebral ischemia. It was pulled out 2 h later to allow reperfusion for 22 h. The cerebral blood flow was measured by rheoencephalograph.

The inclusion criteria for a successful model of CIRI must meet all the following criteria: a) The cerebral blood flow decreased by 50%–60% after 2 h of ischemia and recovered by 30%–40% after reperfusion for 22 h; b) The neurological deficit score (NSS) was between 10 and 12 following 2 h of ischemia. The rats were divided into six groups: a sham group, a model group, several SDSS groups (7.5 mg/kg, 15 mg/kg, 30 mg/kg), and an edaravone group (2.45 mg/kg). SDSS and edaravone were given through tail intravenous injection for 24 h after reperfusion. The rats were then euthanized for further study. Edaravone was used as a positive control due to its efficiency in treating acute ischemic stroke (Xu et al., 2021).

### 2.11 TTC staining

Rats were euthanized by injecting 5% chloral hydrate at 6 ml/kg intraperitoneally. Each rat’s brain tissue was then harvested and coronal sections (without the olfactory bulb, lower brainstem, and cerebellum) were cut at a thickness of 2 mm, 6 pieces in total. These pieces were incubated in 2% TTC solution for 30 min in the dark at 37°C. The samples were then fixed in 4% formaldehyde solution for 24 h. Photographs were taken using a digital camera set at a consistent focal length and brightness. Infarcted brain tissue was identified as pale areas with a diffuse boundary, while normal brain tissue was identified as well-demarcated dark red areas. Image-Pro Plus (IPP) analysis software was used to calculate the sum of infarct volume of the 6 brain pieces, and the relative infarct area was then calculated.

### 2.12 HE staining

The fresh brain tissues were placed in 4% formaldehyde solution for at least 48 h. After dehydration, brain tissues were embedded in paraffin and cut into sections at the thickness of 5 μm. Finally, the sections were stained with hematoxylin and eosin.

### 2.13 Statistical analysis

The results are shown as the means ± SDs. GraphPad Prism 8.0 software was used to carry out one-way analysis of variance (ANOVA) with the least significant difference post hoc test to analyze the data. *p* < 0.05 was considered to indicate statistical significance.

## 3 Results

### 3.1 Sodium danshensu reduces oxygen-glucose deprivation-induced lactate dehydrogenase leakage and cell apoptosis

**FIGURE 1:**
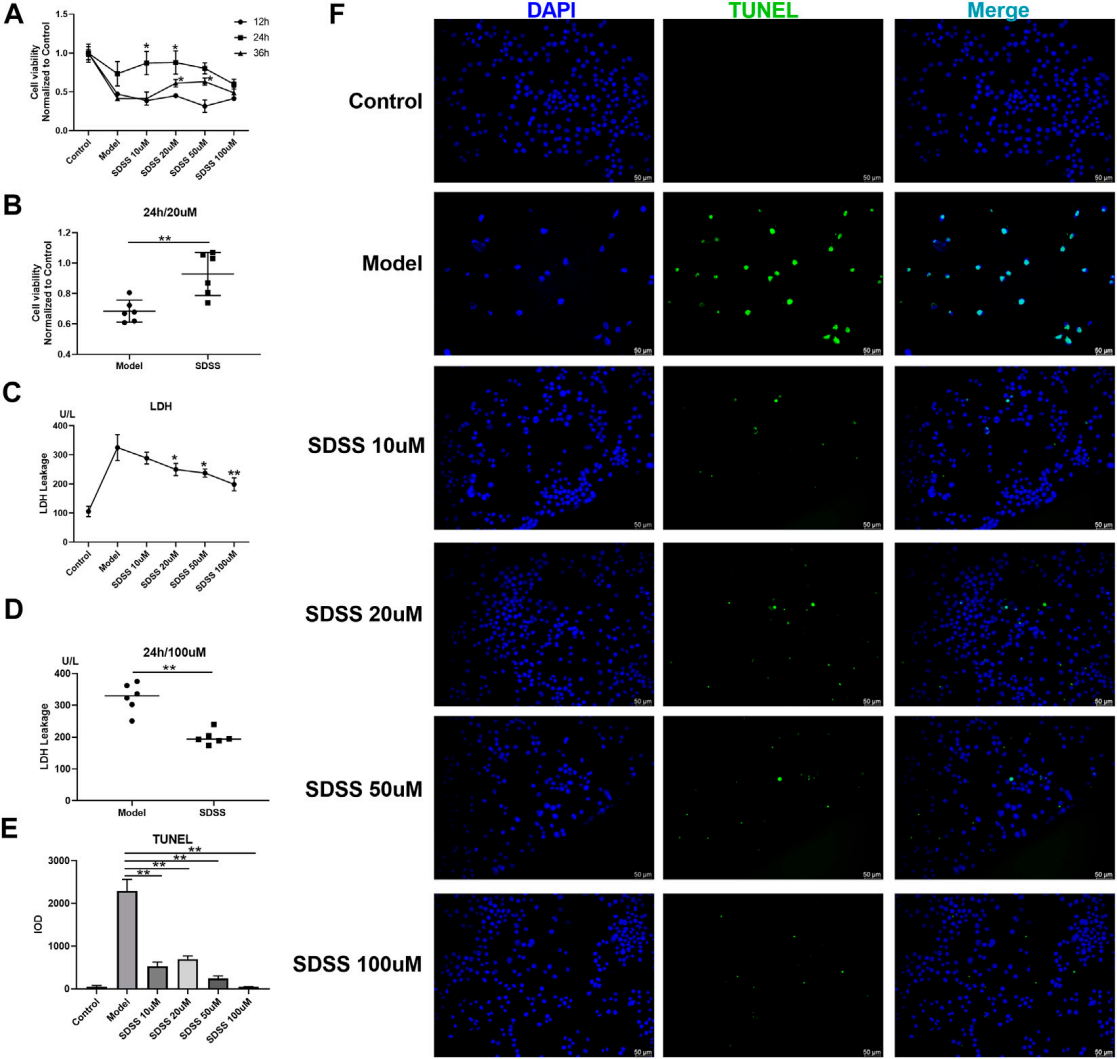
SDSS enhanced cell viability and attenuated ischemia/reperfusion–induced cell apoptosis and LDH release. P12 cell lines were exposed to oxygen-glucose deprivation for 2 h, then were subjected to reoxygenation for either 12 h, 24 h, or 36 h. Cell viability was detected by the CCK8 kit. Cells were treated with 10 μM, 20 μM, 50 μM, or 100 µM SDSS in the reoxygenation stage. The time course of cell viability **(A)** and 20 µM SDSS intervention for 24 h **(B)** in response to ischemia/reperfusion; LDH leakage under different concentrations of SDSS for 24 h; **(C)** LDH leakage following 100 µM SDSS intervention for 24 h **(D)**; **(E)** The integral optical density (IOD) of TUNEL staining. Five images in each group were used to calculate IOD; **(F)** Cell apoptosis rate was detected by TUNEL assay. Number of replicates = 3. Scale bar = 50 µm * = compared to model group, *p* < 0.05; ** = compared to model group, *p* < 0.01.

PC12 cells were exposed to OGD to stimulate ischemia/reperfusion injury. We evaluated the effects of SDSS on cell viability and LDH leakage at the final concentrations of 10 μM, 20 μM, 50 μM, or 100 μM for 12 h, 24 h, and 36 h via the CCK8 test. An elevated level of cell viability after SDSS intervention was confirmed (Figure 1A). The elevated level reached the highest point at 24 h after SDSS intervention in different concentration groups. The most effective concentration of SDSS for increasing cell viability was 20 μM (Figure 1B).

LDH leakage is an important indicator of cell necrosis (Oluyemi et al., 2017). SDSS attenuated OGD-induced LDH leakage in a dose-dependent manner in PC12 cells (Figure 1C). Treatment with 100 μM of SDSS for 24 h significantly reduced the amount of LDH leakage (Figure 1D). TUNEL staining of the PC12 cells showed that OGD-induced apoptosis evidently decreased in all SDSS groups (at concentrations of 10, 20, 50, and 100 μM) (Figures 1E,F). Despite initially being seeded at the same density in each group, the model cells in Figure 1F have a lower seeding density because of anoxia-induced cell death. The above results from *in vitro* studies show the great potential of SDSS for promoting cell viability and reducing apoptosis and necrosis.

### 3.2 AKT1 is a direct binding target of Sodium danshensu

**FIGURE 2:**
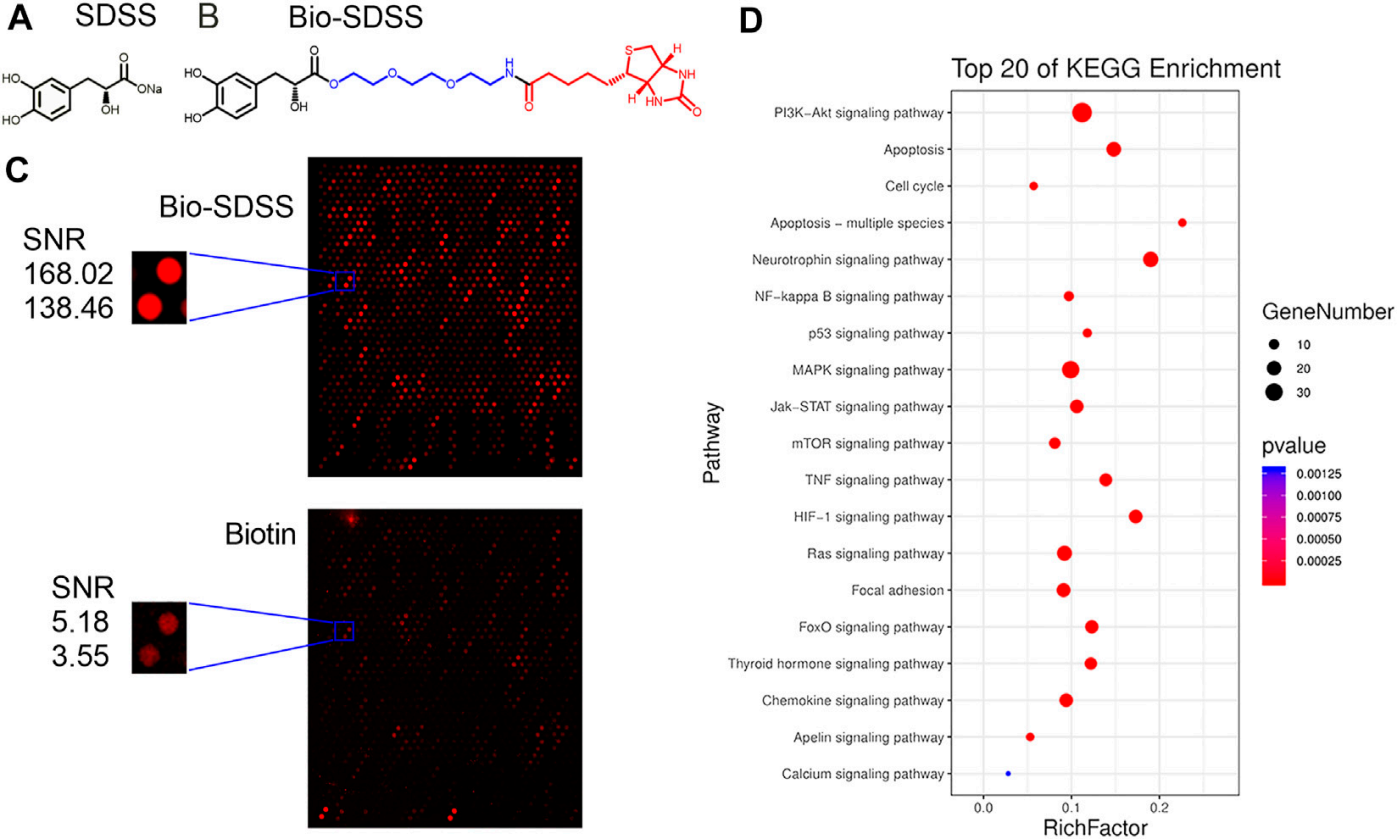
Identification of SDSS binding proteins. **(A)** The chemical structure of SDSS and **(B)** biotin-labeled SDSS (Bio-SDSS). The blue color represents polyethylene glycol (PEG) ligand and the red color represents biotin. **(C)** Representative image of protein array, including positive control spots (Bio-SDSS) and negative control spots (biotin), as well as the magnified image of bio-SDSS binding to AKT1. The signal-to-noise ratio (SNR) is shown. **(D)** The top 20 KEGG enrichment results for SDSS-related targets based on network pharmacology.

The Huprot^TM^ human protein microarray was used to screen the binding proteins of SDSS. First, biotin was used to label SDSS. A polyethylene glycol (PEG) ligand was added in order to increase the water solubility of Bio-SDSS (Figures 2A,B). Bio-SDSS retained the same properties of improving cell viability and reducing LDH leakage as SDSS, as shown in Supplemental Figure S1. Second, both biotin and Bio-SDSS were applied to detect their binding proteins using the HuProt^TM^ human protein microarray. Then, the binding properties were detected by using Cy3-conjugated streptavidin (Figure 2C). In total, 649 proteins were defined as positively binding proteins. The average SNR of Bio-SDSS and biotin with AKT1 and the key regulator of the PI3K-AKT pathway was 153.24 and 4.365, respectively (Figure 2C). Another drug target prediction method, network pharmacology, was also applied to narrow down the scope. The interaction network of the targets of SDSS and the curative targets of CIRI was established using databases including TCMSP, PubChem Compound, SWISS Target, and others (Supplemental Figure S2). Finally, the key targets of SDSS for treating CIRI were confirmed by the intersection of protein microarray results and network pharmacology results (Supplemental Table S1). KEGG enrichment of those interaction targets revealed that the PI3K-AKT signaling pathway was the main pathway of SDSS in treating CIRI (Figure 2D).

### 3.3 Sodium danshensu activates AKT1 phosphorylation in PC12 and HAPI cell lines

**FIGURE 3:**
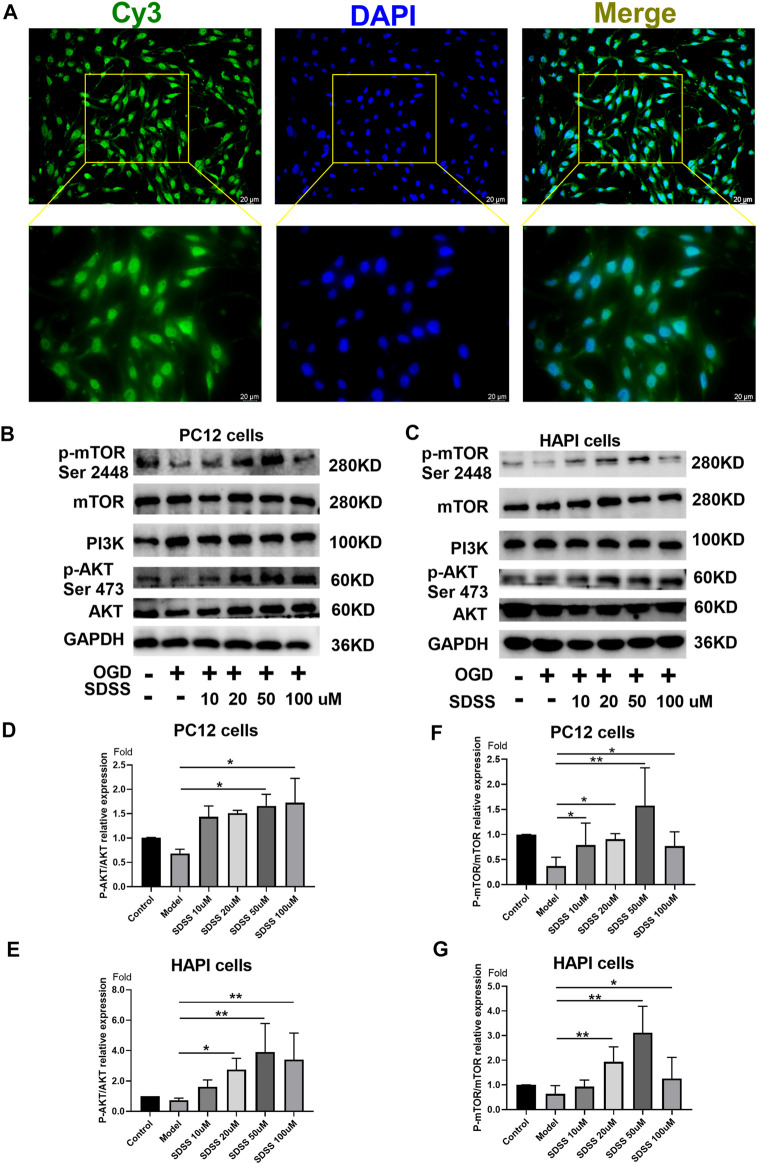
SDSS is localized in the cell and activates AKT1 phosphorylation. **(A)** Bio-SDSS distribution in HAPI cells. Expression levels of p-mTOR (ser2448), PI3K, and P-AKT in PC12 cells **(B)** and HAPI cells **(C)**, as shown by Western blot. Quantification of the expression levels of p-mTOR (ser2448) **(D)** and p-AKT **(E)** in PC12 cells. Quantification of the expression levels of p-mTOR (ser2448) **(F)** and p-AKT **(G)** in HAPI cells. Number of replicates = 3. * = *p* < 0.05; ** = *p* < 0.01.

The distribution of Bio-SDSS in cells was detected by Cy3-conjugated streptavidin, which tightly binds biotin. Green fluorescence can be detected in both cytoplasm and nucleus, which indicates that Bio-SDSS can pass through cytomembrane and karyotheca (Figure 3A). We then determined the effect of SDSS on the expression or phosphorylation level of AKT1, as well as its upstream and downstream regulators, PI3K and mTOR, in both PC12 and HAPI cell lines (Figures 3B,C). Phosphorylated AKT1was increased upon SDSS administration, in a dose-dependent manner, in these two cell lines (Figures 3D,E). Moreover, the level of phosphorylated mTOR was increased following SDSS intervention (Figures 3F,G). However, the expression level of PI3K did not change in either the PC12 or HAPI cell lines (Supplemental Figure S3). These results indicate that SDSS could influence the downstream regulator of AKT1 but not the upstream regulator, which implies AKT1 is the potential target of SDSS.

### 3.4 Binding sites of sodium danshensu to AKT1 protein

**FIGURE 4:**
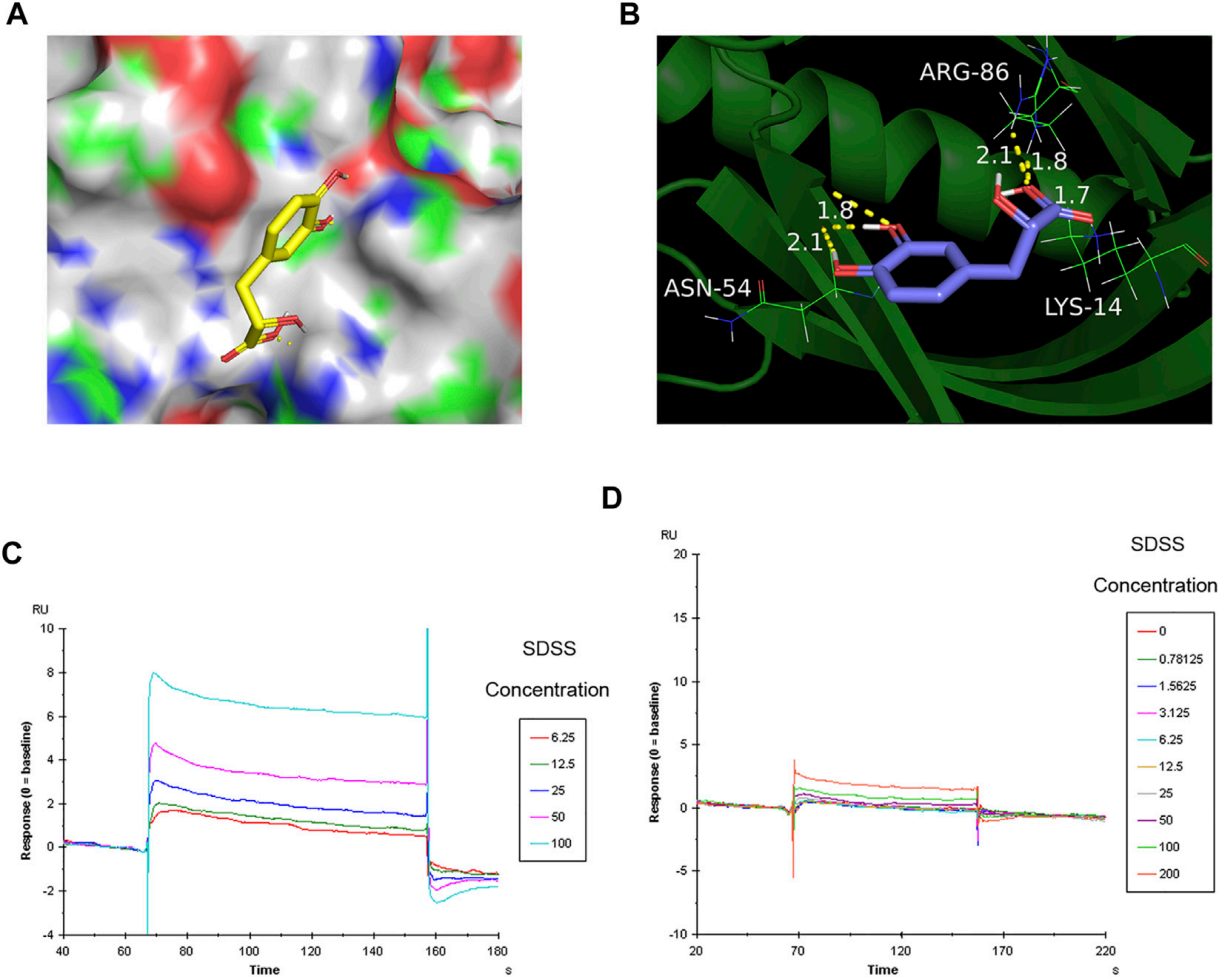
The binding sites of SDSS to AKT1 were predicted by Autodock and then verified by SPR. **(A)** The 3D structure of SDSS docked by the AKT1 PH domain. SDSS is shown in sticks mode; AKT1 is shown in color surface mode. **(B)** The binding site of SDSS with the AKT1 PH domain. SDSS is shown in sticks mode; AKT1 is shown in cartoon mode. Surface plasmon resonance (SPR) analysis showing a direct interaction between SDSS and wide-type AKT1 **(C)** or mutant AKT1 **(D)**. The Y-axis represents the response value between SDSS and proteins. The X-axis represents the reaction time between SDSS and proteins. The unit used for SDSS concentration was µM.

**FIGURE 5:**
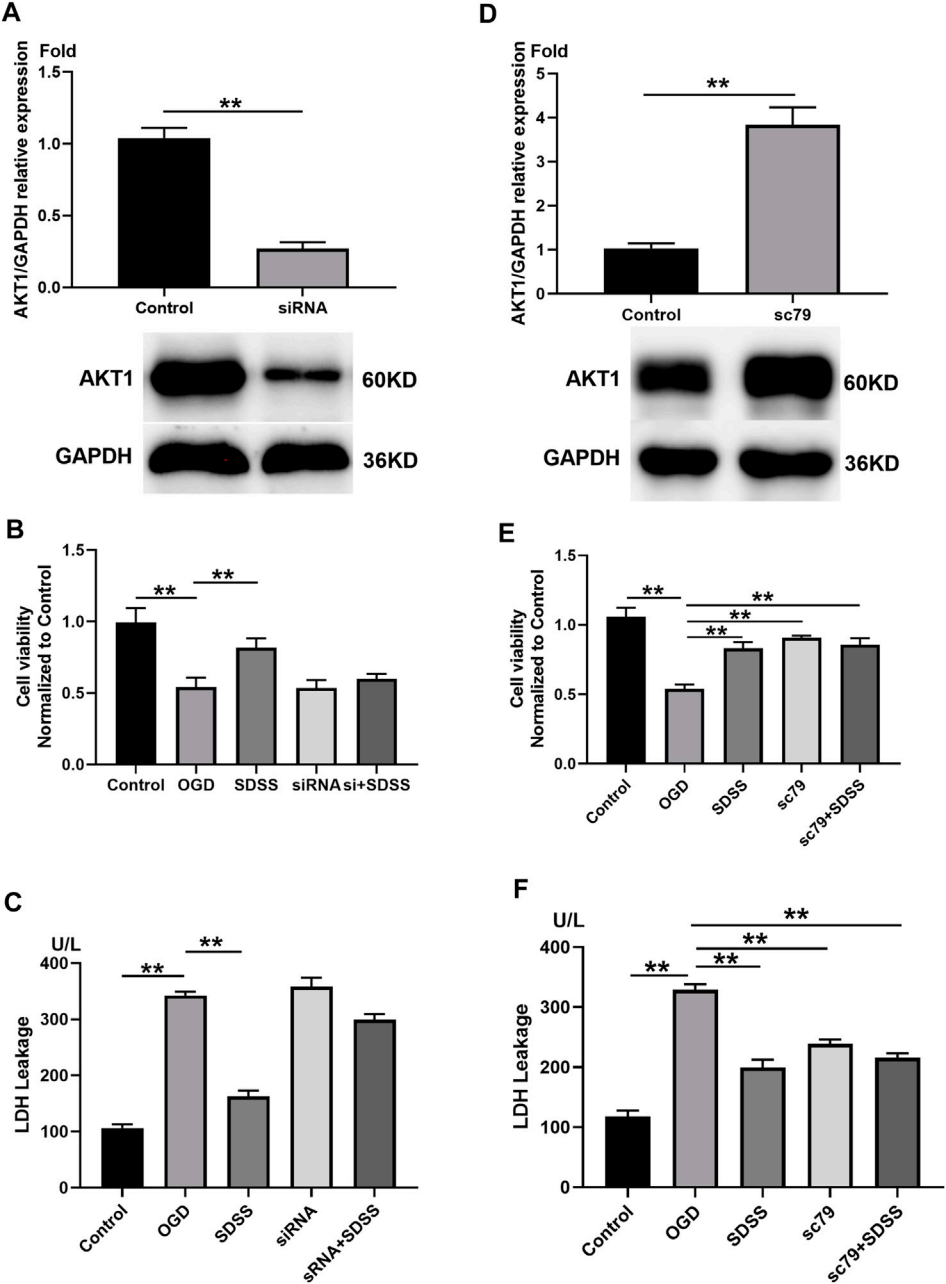
The role of AKT1 in SDSS-mediated neuron protection. **(A)** PC12 cells were transfected with siRNA that targeted AKT1. Control cells were transfected with negative control siRNA. The expression level of AKT1 in PC12 cells upon siRNA treatment was shown by Western blot. Analysis of cell viability **(B)** and LDH release **(C)** following AKT1 knockdown. **(D)** PC12 cells were treated with AKT1 activator SC79. Control cells were treated with DMSO. The expression level of AKT1 in PC12 cells following the addition of SC79 or DMSO. Analysis of cell viability **(E)** and LDH release **(F)** following AKT1 overexpression. For all Western blot experiments, number of replicates = 3. * = *p* < 0.05; ** = *p* < 0.01.

To further confirm the binding of SDSS to AKT1 protein, Autodock 4.1 software (Goodsell et al., 2020) was used to predict the binding sites. As shown by the software, SDSS was docked by the PH domain of AKT1 at sites ASN-54, ARG-86, and LYS-14 (Figures 4A,B). The results of the SPR analysis showed a direct interaction between SDSS and wide-type AKT1 as the binding energy increased in a concentration-dependent manner between 0 uM and 200 uM (Figure 5C). However, mutant AKT1 (Mut-K14R-R86H-N54Q) failed to bind SDSS, as the binding energy did not increase along with higher mutant AKT1 concentrations (Figure 5D). SC79, a well-known AKT1 activator that served as a positive control, directly interacts with wide-type rat AKT1 protein (Supplemental Figure S4) (Liu et al., 2018). Unfortunately, the response signal between SDSS and AKT1 protein did not reach a saturation state, therefore the dissociation constant (KD) value cannot be calculated.

### 3.5 Sodium danshensu attenuates the decreased viability and lactate dehydrogenase leakage induced by oxygen-glucose deprivation *via* targeting AKT1

siRNA treatment significantly knocked down the expression of AKT1 (Figure 5A), which blocked the role of SDSS in restoring cell viability (Figure 5B) and attenuating LDH leakage stemming from the OGD state (Figure 5C). By contrast, the addition of SC79 upregulated AKT1 (Figure 5D), but did not alter the role of SDSS in reviving neurons and preventing LDH leakage. Through the overexpression and the knockout of AKT1 protein expression *in vitro*, we further confirm that SDSS plays a significant role in regulating AKT1 activation.

### 3.6 Administration of SDSS reduces ischemia/reperfusion–induced brain necrosis and apoptosis

**FIGURE 6:**
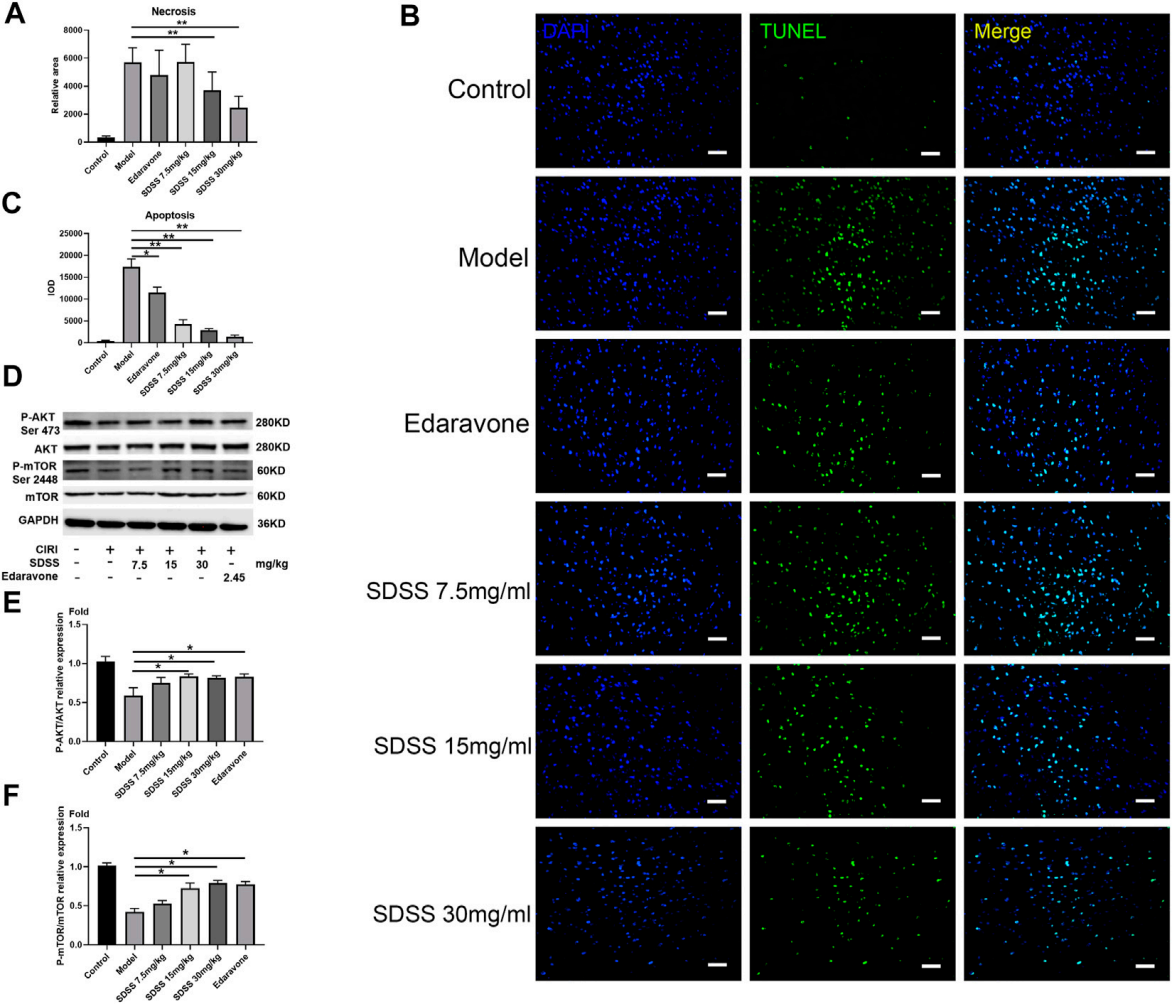
SDSS prevents apoptosis in rats after CIRI. **(A)** The necrosis area (calculated from Figure 6D) in the brain tissue. **(B)** TUNEL staining of brain tissues after ischemia/reperfusion in each group. **(C)** Apoptosis level in the brain samples. **(D)** Expression levels of p-mTOR (ser2448) and p-AKT (ser473) in the brain samples, as shown by Western blot. Quantification of the expression levels of **(E)** p-AKT (ser473) and **(F)** p-mTOR (ser2448) in brain tissues. Number of replicates = 3. * = *p* < 0.05; ** = *p* < 0.01.

Figure 6A displays the operative site used on the mice under study. Figure 6B shows that bloodstreams in states of ischemia and reperfusion were at 40%–50% and 60%–70%, respectively, compared to their normal state. SDSS at 7.5 mg/kg, 15 mg/kg, or 30 mg/kg, along with edaravone, were administrated through tail vein injection. Compared to the control group, cell arrangement was disordered and there was a marked increase in cellular edema in the brain tissues of the model group. Administration of both SDSS (at 7.5 mg/kg, 15 mg/kg, or 30 mg/kg) and edaravone attenuated pathological changes in the SDSS intervention groups (Figure 6C).

**FIGURE 7:**
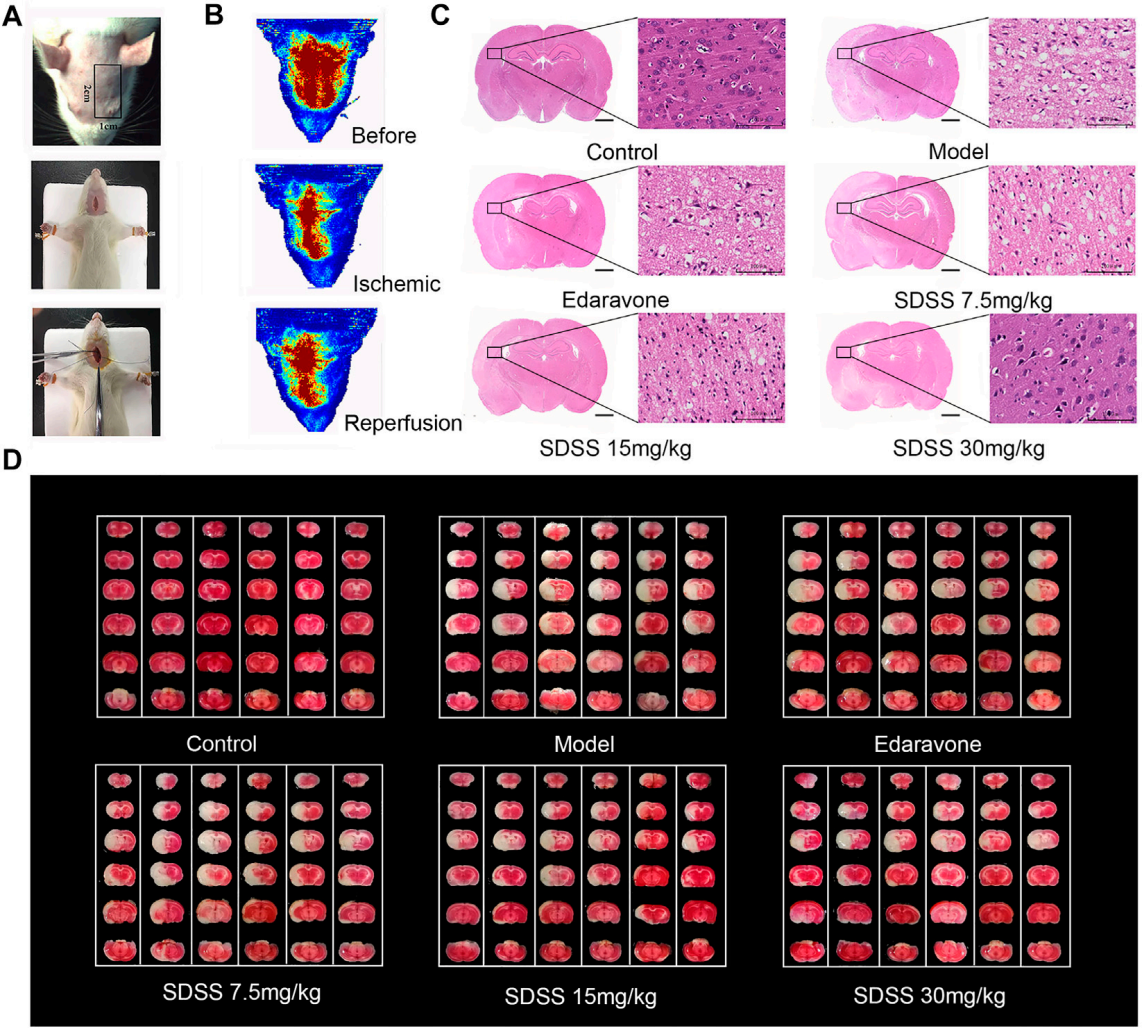
SDSS alleviates CIRI in rats. **(A)** Operation procedures for the CIRI model. The first row shows the detection area of the bloodstream. The second and third rows show the insertion of intraluminal thread. **(B)** Representations of rheoencephalograms of rats in healthy, ischemic, and reperfusion stages. **(C)** HE staining of the brain tissue samples in control, ischemia/reperfusion model, and SDSS cohorts. **(D)** TTC staining of whole brain tissue after ischemia/reperfusion in each group. The white areas represent necrosis. For all HE and TTC staining experiments, each lane contains one whole brain tissue sample, which has been cut to 6 pieces in order to do TTC staining. Number of replicates = 6.

Moreover, both 15 mg/kg and 30 mg/kg SDSS administrations led to a better outcome than that of edaravone alone. As shown in Figure 6D, the necrosis area was markedly increased in the model group compared with the control group, while treatment with both SDSS (at 7.5 mg/kg, 15 mg/kg, or 30 mg/kg) and edaravone significantly reduced the necrosis area from the IR background. The necrosis rate in two of the SDSS groups (15 mg/kg and 30 mg/kg) was markedly reduced compared to that of the model group (Figure 7A).

Similarly, TUNEL staining showed that the apoptosis rate increased significantly in the model group and was attenuated by administration of SDSS (at 7.5 mg/kg, 15 mg/kg, or 30 mg/kg) or edaravone (Figures 7A,C). Furthermore, the expressions of pAKT1/AKT1 and p-mTOR/mTOR were markedly decreased compared to those in the control group, but significantly increased in the SDSS groups compared to the model group (Figures 7D–F). To sum up, our *in vivo* studies showed that SDSS attenuated CIRI through promoting AKT1 activation.

## 4 Discussion

Several studies have reported that SDSS exerts neuroprotective effects, possibly through activating the PI3K/AKT pathway. In mice models of Parkinson’s disease, SDSS notably increased the expression of p-PI3K and p-AKT (Tian et al., 2020). Furthermore, in rat models of cerebral ischemia and reperfusion injury, SDSS treatment also increased the level of p-AKT (Guo et al., 2015).

Our study also demonstrates that SDSS administration protects against CIRI-induced necrosis and apoptosis both *in vivo* and *in vitro*. We set up several drug concentrations and time frames in order to observe whether SDSS promotes cell viability in a dose- or time-dependent manner. However, neither a dose- nor time-dependent paradigm was evident. We therefore chose the best concentration and incubation time of SDSS for further study, namely, 20 μM and incubation for 24 h. Similar results have been reported for edaravone, a free radical scavenger that is clinically approved for the treatment of acute ischemic strokes. Cells showed the greatest viability with an edaravone concentration of 25 μM; above this concentration, cell viability declined (Shou et al., 2019).

Additionally, AKT serine phosphorylation is upregulated upon SDSS intervention, and its downstream regulator, mTOR, is also activated (but not its upstream activator, PI3K). mTOR is critical for cellular growth, proliferation, and metabolism (Li et al., 2018). Once mTOR is activated, it relieves necrosis and apoptosis (Shi et al., 2019; Zhang et al., 2020). The AKT/mTOR pathway is therefore highly activated in numerous cancers (Izzedine et al., 2013). Our results show that SDSS localizes in both the cytoplasm and nucleus, which is a prerequisite for SDSS playing a role in protein interaction and gene expression. We also prove that AKT knockdown reduces the protective effects of SDSS against CIRI, which indicates AKT is a potential target for SDSS in treating CIRI.

As a protein serine/threonine kinase, AKT has three enzymatic isoforms, termed AKT1, AKT2, and AKT3 (Yu et al., 2021). Although these three isoforms are of similar substrate specificity, they are different in their tissue specificity in adults: AKT1 is abundant in the brain, heart, and lungs; AKT2 is most prevalent in skeletal muscle; AKT3 is prevalent in the brain and kidney tissues (Shiojima and Walsh, 2002; Paez and Sellers, 2003; Hui et al., 2005). AKT activation attenuates ischemic reperfusion injury in other tissues, such as those of the kidneys, liver, and heart (Kandel and Hay, 1999; Chen et al., 2020; Lin et al., 2021). Based on the results of the Huprot^TM^ human protein microarray, AKT1 and AKT2 had high SNR signals compared with AKT3. Since the main AKT isoform in the brain is AKT1, we mainly focused on this isoform.

Autodock software was used to predict the binding sites of SDSS and AKT1. AKT has an N-terminal pleckstrin homology (PH) domain, a central catalytic domain, and a C-terminal regulatory domain (Cai and Semenza, 2004). Our results showed that SDSS was perfectly docked in the PH domain of AKT1, and the binding sites were ASN-54, ARG-86, and LYS-14 (K14-R86-N54). Then, as shown in the SPR experiment, SDSS bound to wide-type AKT1 but not mutant AKT1 (Mut-K14R-R86H-N54Q). Protein mutation is very difficult to prepare *in vitro* because the amino acid exchange sometimes has a big influence on the protein tertiary structure (Fang, 2019). We only successfully synthesized one type of mutant AKT1 protein, while another mutant type (Mut-K14R-R86K-N54Q) failed due to low protein concentration. Although SC79 has been widely used commercially as an AKT activator, its binding sites with AKT have not yet been clarified (Fang, 2019). It has been reported that the specific binding sites of small molecules and proteins can be further confirmed by x-ray diffraction analysis and/or cryo-electron microscopy (Jo et al., 2012). At any rate, we can confirm here that SDSS can bind to AKT1, and that the amino acids ASN-54, ARG-86 and LYS-14 are the likely binding sites.

The PH domain is important for the activation of AKT because it binds to the membrane phospholipid phosphoinositol-(3,4,5)-phosphate (PIP3) and locates AKT to the plasma membrane (Fan et al., 2020). Translocation from the cytoplasm to the membrane is necessary for AKT activation. Before translocation, it has to be phosphorylated by phosphoinositide-dependent kinase-1 (PDK1) at site Thr308 and by mTOR (Truebestein et al., 2021) at site Ser473. Therefore, translocation and phosphorylation are the two steps responsible for AKT activation (Kearney et al., 2021). It has been reported that phosphorylation of S473 is essential for AKT activation through the formation of an electrostatic interaction in the PH-kinase domain linker, thereby relieving PH domain–mediated autoinhibition (Chu et al., 2020). We can therefore detect the AKT Ser473 phosphorylation level after SDSS intervention. One drawback is that the phosphorylation status of Thr308 residue, which is also very important for the full activation of AKT, is not detected (Balasuriya et al., 2020). Our results demonstrate that SDSS enhances the phosphorylation level of AKT1, but whether SDSS participated in the AKT1 translocation process is still to be elucidated. Since the PH domain is highly conserved among all three isoforms of AKT (Gerta et al., 2020; Stocker et al., 2002), it is reasonable to assume that SDSS could prevent ischemic reperfusion injury in other tissues, like those of the kidneys, liver, etc.

Sodium Danshensu, also called 3,4-dihydroxy phenyl lactic acid, can be found in many naturally growing plants and is easily purified (Solomon, 2018). Its pharmacological activities, such as antioxidation, anti-apoptosis, and anti-inflammation, have been well studied (Zhang et al., 2019). For these reasons, SDSS is widely used in the treatment of cardiovascular and cerebral diseases in China (Li et al., 2018). Our previous study demonstrated the cardioprotective effect of SDSS in isolated hearts suffering from ischemic reperfusion injury using a Langendorff apparatus (Gao et al., 2017). The results of that study showed a decreased rate of apoptosis in cardiomyocytes in the SDSS group, but whether AKT1 was one of the targets was not clarified. A toxicological study indicated that SDSS had no adverse effect at doses of 50 mg/kg, 150 mg/kg, and 450 mg/kg for 90 days (Gao et al., 2009), confirming its safety.

The present study confirms that AKT1 is one of the targets of SDSS in treating CIRI. SDSS activated AKT1 by binding to its PH domain, given that ASN-54, ARG-86, and LYS-14 are all located there. SDSS also promoted the AKT1 phosphorylation rate, as well as that of its downstream regulator, ultimately leading to a decrease in apoptosis and necrosis of brain tissues. Although there are 649 proteins in total that bind to SDSS, according to the results of the HuProt^TM^ human protein microarray, we only examined the proteins ranked first in the KEGG pathways according to network pharmacology. Other proteins may also be targets of SDSS, and this needs to be further elucidated.

## 5 Conclusion

SDSS enhanced cell viability and attenuated apoptosis after ischemia and reperfusion injury in the brain, both *in vivo* and *in vitro*. SDSS targets the PH domain of AKT1, at binding sites ASN-53, ARG-86, and LYS-14. AKT1 knockdown abolished the role of SDSS in activating AKT1. In summary, the present study provides evidence that SDSS attenuates CIRI injury by binding and activating AKT1, thereby relieving the necrosis and apoptosis rate of brain tissues (; Chu et al., 2015; Liu et al., 2018; Meschia and Brott, 2018).

## Data Availability

The original contributions presented in the study are included in the article/Supplementary Materials; further inquiries can be directed to the corresponding authors.
