# Supplementary material for: Sodium danshensu attenuates cerebral ischemia–reperfusion injury by targeting AKT1
Source: Front Pharmacol. 2022 Sep 15;13:946668. doi: 10.3389/fphar.2022.946668 (PMC9520076; doi:10.3389/fphar.2022.946668)
Supplement: Supplementary file 1 [file DataSheet5.doc]

**Supplemental material**

# **Supplemental material I. Information about Human recombinant wild type and mutant AKT1 protein**

# Homo sapiens v-akt murine thymoma viral oncogene homolog 1, mRNA (cDNA clone MGC:8686 IMAGE:2964603), complete cds

GenBank: BC000479.2


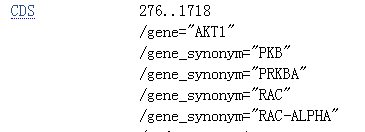


**Wide type AKT1**

**Protein sequence：**

Has-AKT1-WT （1443bp）

MSDVAIVKEGWLH**K**RGEYIKTWRPRYFLLKNDGTFIGYKERPQDVDQREAPLN**N**FSVAQCQLMKTERPRPNTFIIRCLQWTTVIE**R**TFHVETPEEREEWTTAIQTVADGLKKQEEEEMDFRSGSPSDNSGAEEMEVSLAKPKHRVTMNEFEYLKLLGKGTFGKVILVKEKATGRYYAMKILKKEVIVAKDEVAHTLTENRVLQNSRHPFLTALKYSFQTHDRLCFVMEYANGGELFFHLSRERVFSEDRARFYGAEIVSALDYLHSEKNVVYRDLKLENLMLDKDGHIKITDFGLCKEGIKDGATMKTFCGTPEYLAPEVLEDNDYGRAVDWWGLGVVMYEMMCGRLPFYNQDHEKLFELILMEEIRFPRTLGPEAKSLLSGLLKKDPKQRLGGGSEDAKEIMQHRFFAGIVWQHVYEKKLSPPFKPQVTSETDTRYFDEEFTAQMITITPPDQDDSMECVDSERRPHFPQFSYSASGTA

**Nucleic acid sequence：**ATGAGCGACGTGGCTATTGTGAAGGAGGGTTGGCTGCAC**AAA**CGAGGGGAGTACATCAAGACCTGGCGGCCACGCTACTTCCTCCTCAAGAATGATGGCACCTTCATTGGCTACAAGGAGCGGCCGCAGGATGTGGACCAACGTGAGGCTCCCCTCAAC**AAC**TTCTCTGTGGCGCAGTGCCAGCTGATGAAGACGGAGCGGCCCCGGCCCAACACCTTCATCATCCGCTGCCTGCAGTGGACCACTGTCATCGAA**CGC**ACCTTCCATGTGGAGACTCCTGAGGAGCGGGAGGAGTGGACAACCGCCATCCAGACTGTGGCTGACGGCCTCAAGAAGCAGGAGGAGGAGGAGATGGACTTCCGGTCGGGCTCACCCAGTGACAACTCAGGGGCTGAAGAGATGGAGGTGTCCCTGGCCAAGCCCAAGCACCGCGTGACCATGAACGAGTTTGAGTACCTGAAGCTGCTGGGCAAGGGCACTTTCGGCAAGGTGATCCTGGTGAAGGAGAAGGCCACAGGCCGCTACTACGCCATGAAGATCCTCAAGAAGGAAGTCATCGTGGCCAAGGACGAGGTGGCCCACACACTCACCGAGAACCGCGTCCTGCAGAACTCCAGGCACCCCTTCCTCACAGCCCTGAAGTACTCTTTCCAGACCCACGACCGCCTCTGCTTTGTCATGGAGTACGCCAACGGGGGCGAGCTGTTCTTCCACCTGTCCCGGGAGCGTGTGTTCTCCGAGGACCGGGCCCGCTTCTATGGCGCTGAGATTGTGTCAGCCCTGGACTACCTGCACTCGGAGAAGAACGTGGTGTACCGGGACCTCAAGCTGGAGAACCTCATGCTGGACAAGGACGGGCACATTAAGATCACAGACTTCGGGCTGTGCAAGGAGGGGATCAAGGACGGTGCCACCATGAAGACCTTTTGCGGCACACCTGAGTACCTGGCCCCCGAGGTGCTGGAGGACAATGACTACGGCCGTGCAGTGGACTGGTGGGGGCTGGGCGTGGTCATGTACGAGATGATGTGCGGTCGCCTGCCCTTCTACAACCAGGACCATGAGAAGCTTTTTGAGCTCATCCTCATGGAGGAGATCCGCTTCCCGCGCACGCTTGGTCCCGAGGCCAAGTCCTTGCTTTCAGGGCTGCTCAAGAAGGACCCCAAGCAGAGGCTTGGCGGGGGCTCCGAGGACGCCAAGGAGATCATGCAGCATCGCTTCTTTGCCGGTATCGTGTGGCAGCACGTGTACGAGAAGAAGCTCAGCCCACCCTTCAAGCCCCAGGTCACGTCGGAGACTGACACCAGGTATTTTGATGAGGAGTTCACGGCCCAGATGATCACCATCACACCACCTGACCAAGATGACAGCATGGAGTGTGTGGACAGCGAGCGCAGGCCCCACTTCCCCCAGTTCTCCTACTCGGCCAGCGGCACGGCCTGA

**Mutant AKT1**

Has-AKT1-Mut- K14R- R86H- N54Q

**Protein sequence**：MSDVAIVKEGWLH**R**RGEYIKTWRPRYFLLKNDGTFIGYKERPQDVDQREAPLN**Q**FSVAQCQLMKTERPRPNTFIIRCLQWTTVIE**H**TFHVETPEEREEWTTAIQTVADGLKKQEEEEMDFRSGSPSDNSGAEEMEVSLAKPKHRVTMNEFEYLKLLGKGTFGKVILVKEKATGRYYAMKILKKEVIVAKDEVAHTLTENRVLQNSRHPFLTALKYSFQTHDRLCFVMEYANGGELFFHLSRERVFSEDRARFYGAEIVSALDYLHSEKNVVYRDLKLENLMLDKDGHIKITDFGLCKEGIKDGATMKTFCGTPEYLAPEVLEDNDYGRAVDWWGLGVVMYEMMCGRLPFYNQDHEKLFELILMEEIRFPRTLGPEAKSLLSGLLKKDPKQRLGGGSEDAKEIMQHRFFAGIVWQHVYEKKLSPPFKPQVTSETDTRYFDEEFTAQMITITPPDQDDSMECVDSERRPHFPQFSYSASSTA

**Nucleic acid sequence**：

ATGAGCGACGTGGCTATTGTGAAGGAGGGTTGGCTGCAC**CGT**CGAGGGGAGTACATCAAGACCTGGCGGCCACGCTACTTCCTCCTCAAGAATGATGGCACCTTCATTGGCTACAAGGAGCGGCCGCAGGATGTGGACCAACGTGAGGCTCCCCTCAAC**CAA**TTCTCTGTGGCGCAGTGCCAGCTGATGAAGACGGAGCGGCCCCGGCCCAACACCTTCATCATCCGCTGCCTGCAGTGGACCACTGTCATCGAA**CAT**ACCTTCCATGTGGAGACTCCTGAGGAGCGGGAGGAGTGGACAACCGCCATCCAGACTGTGGCTGACGGCCTCAAGAAGCAGGAGGAGGAGGAGATGGACTTCCGGTCGGGCTCACCCAGTGACAACTCAGGGGCTGAAGAGATGGAGGTGTCCCTGGCCAAGCCCAAGCACCGCGTGACCATGAACGAGTTTGAGTACCTGAAGCTGCTGGGCAAGGGCACTTTCGGCAAGGTGATCCTGGTGAAGGAGAAGGCCACAGGCCGCTACTACGCCATGAAGATCCTCAAGAAGGAAGTCATCGTGGCCAAGGACGAGGTGGCCCACACACTCACCGAGAACCGCGTCCTGCAGAACTCCAGGCACCCCTTCCTCACAGCCCTGAAGTACTCTTTCCAGACCCACGACCGCCTCTGCTTTGTCATGGAGTACGCCAACGGGGGCGAGCTGTTCTTCCACCTGTCCCGGGAGCGTGTGTTCTCCGAGGACCGGGCCCGCTTCTATGGCGCTGAGATTGTGTCAGCCCTGGACTACCTGCACTCGGAGAAGAACGTGGTGTACCGGGACCTCAAGCTGGAGAACCTCATGCTGGACAAGGACGGGCACATTAAGATCACAGACTTCGGGCTGTGCAAGGAGGGGATCAAGGACGGTGCCACCATGAAGACCTTTTGCGGCACACCTGAGTACCTGGCCCCCGAGGTGCTGGAGGACAATGACTACGGCCGTGCAGTGGACTGGTGGGGGCTGGGCGTGGTCATGTACGAGATGATGTGCGGTCGCCTGCCCTTCTACAACCAGGACCATGAGAAGCTTTTTGAGCTCATCCTCATGGAGGAGATCCGCTTCCCGCGCACGCTTGGTCCCGAGGCCAAGTCCTTGCTTTCAGGGCTGCTCAAGAAGGACCCCAAGCAGAGGCTTGGCGGGGGCTCCGAGGACGCCAAGGAGATCATGCAGCATCGCTTCTTTGCCGGTATCGTGTGGCAGCACGTGTACGAGAAGAAGCTCAGCCCACCCTTCAAGCCCCAGGTCACGTCGGAGACTGACACCAGGTATTTTGATGAGGAGTTCACGGCCCAGATGATCACCATCACACCACCTGACCAAGATGACAGCATGGAGTGTGTGGACAGCGAGCGCAGGCCCCACTTCCCCCAGTTCTCCTACTCGGCCAGCGGCACGGCCTGA
