# Supplementary figures and images for: Sodium danshensu attenuates cerebral ischemia–reperfusion injury by targeting AKT1
Source: Front Pharmacol. 2022 Sep 15;13:946668. doi: 10.3389/fphar.2022.946668 (PMC9520076; doi:10.3389/fphar.2022.946668)

**Supplemental** **Figure IV.** SPR analysis of the interaction between sc79 and recombinant rat AKT1.


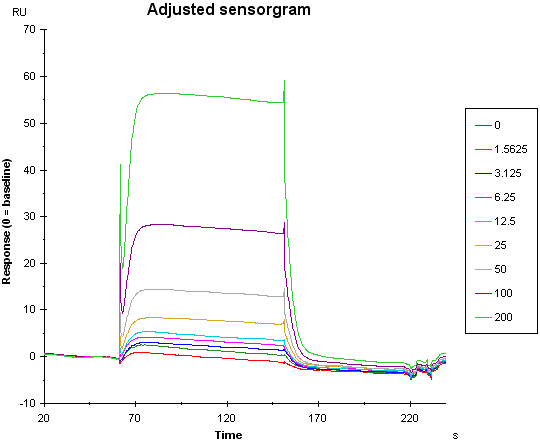

Supplement: Supplementary file 2 [file DataSheet4.doc]
