# Supplementary material for: Sodium danshensu attenuates cerebral ischemia–reperfusion injury by targeting AKT1
Source: Front Pharmacol. 2022 Sep 15;13:946668. doi: 10.3389/fphar.2022.946668 (PMC9520076; doi:10.3389/fphar.2022.946668)

**Supplemental Figure III.** Quantification of the expression levels of PI3K in PC12 cells (A) and HAPI cells (B). Number of replicates =3.


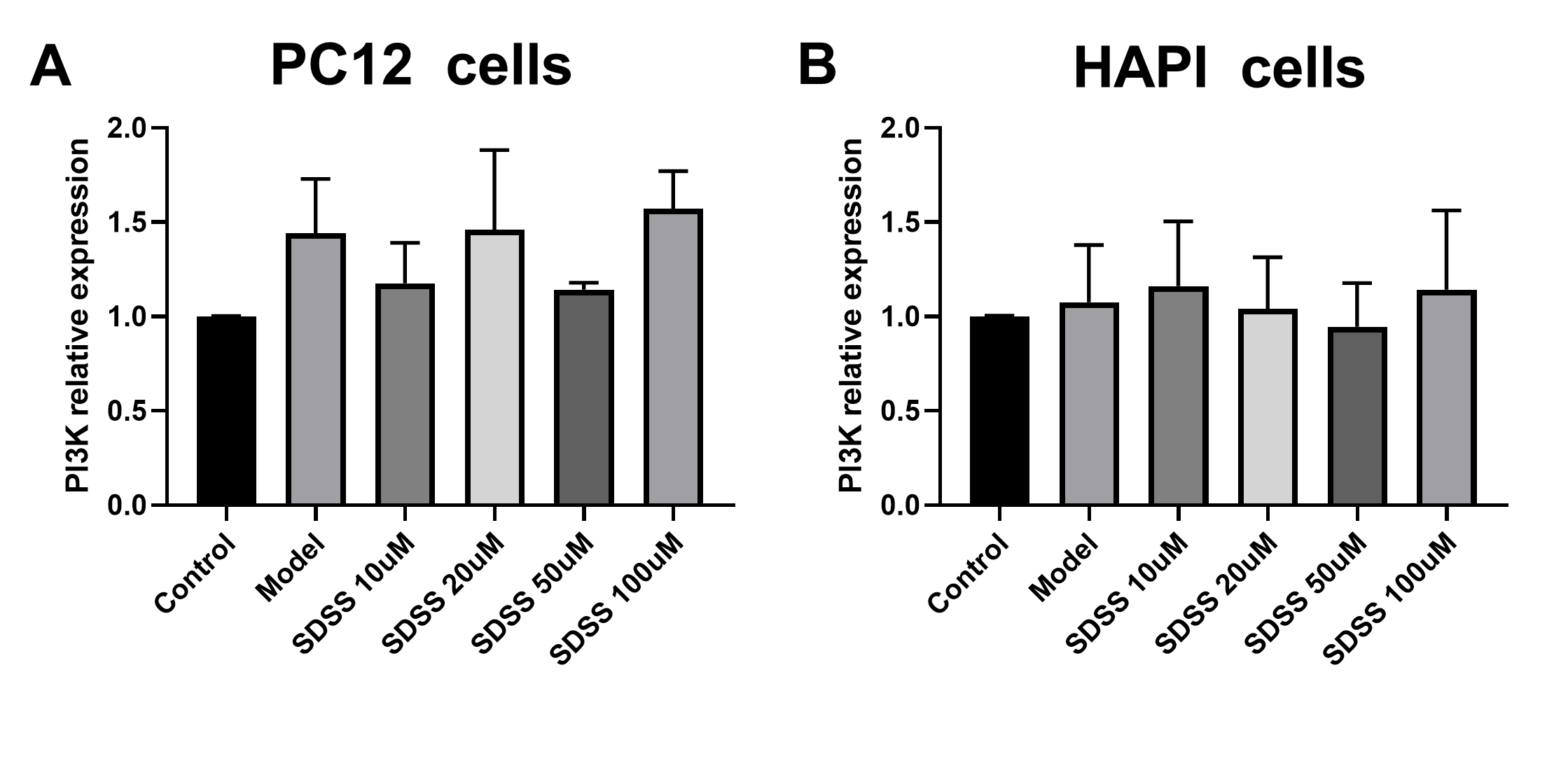

Supplement: Supplementary file 3 [file DataSheet3.doc]
