# Supplementary material for: Sodium danshensu attenuates cerebral ischemia–reperfusion injury by targeting AKT1
Source: Front Pharmacol. 2022 Sep 15;13:946668. doi: 10.3389/fphar.2022.946668 (PMC9520076; doi:10.3389/fphar.2022.946668)

**Supplemental Figure II.** (A) The network of interaction between CIRI and related targets; (B)The network of interaction between SDSS and related targets.


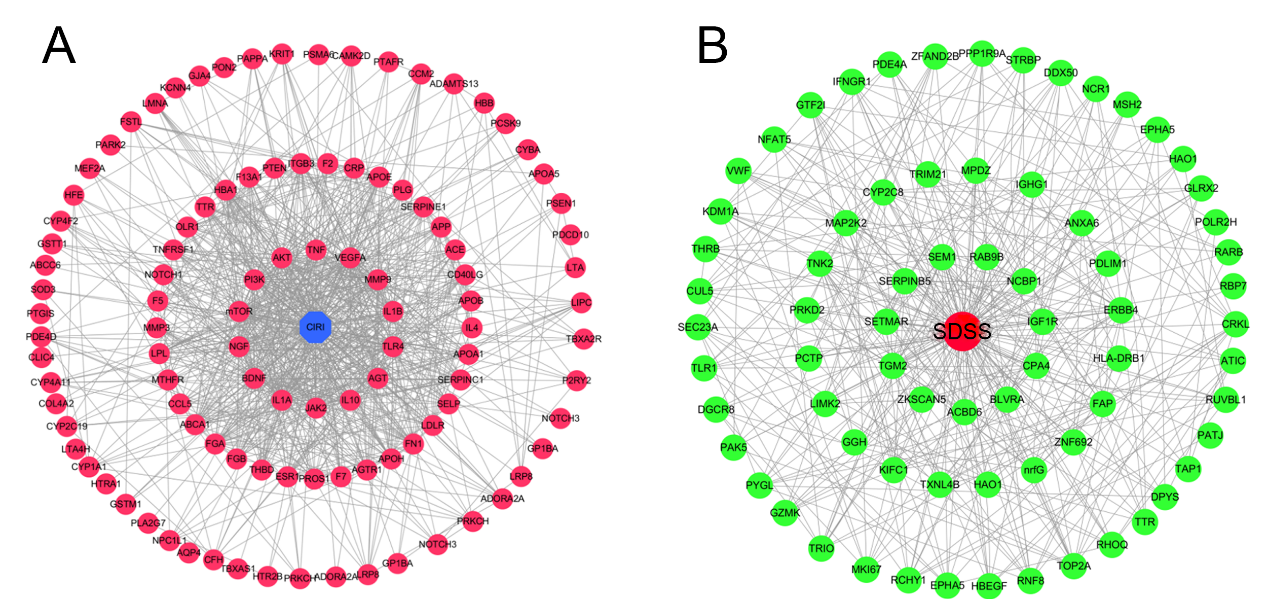

Supplement: Supplementary file 4 [file DataSheet2.doc]
